# Assessment of health problems of sheep and goats based on ante-mortem and post-mortem inspection at Addis Ababa Abattoir, Ethiopia

**DOI:** 10.3389/fvets.2024.1406801

**Published:** 2024-06-06

**Authors:** Tizeta Bekele Atoma, Barbara Szonyi, Aklilu Feleke Haile, Reinhard Fries, Maximillian P. O. Baumann, Delia Grace Randolph

**Affiliations:** ^1^International Livestock Research Institute (ILRI), Addis Ababa, Ethiopia; ^2^Aklilu Lemma Institute of Pathobiology, Addis Ababa University, Addis Ababa, Ethiopia; ^3^Institute of Meat Hygiene and Technology, FAO Reference Centre for Veterinary Public Health (VPH), Freie Universität Berlin, Berlin, Germany; ^4^International Livestock Research Institute (ILRI), Nairobi, Kenya

**Keywords:** Ethiopia, goats, lesion, meat inspection, parasites, sheep, small ruminants

## Abstract

**Introduction:**

Ethiopia has a rapidly growing small ruminant sector, which faces low productivity due to husbandry practices and poor health condition of the animals. A study was conducted in Ethiopia’s largest municipal abattoir with the objective to assess the health problems of sheep and goats presented for slaughter using standard ante-mortem and post-mortem methodology.

**Methods:**

A cross-sectional study using systematic random sampling was conducted on 384 sheep and 384 goats from January to July 2014.

**Results:**

Soiled skin (69.1%), poor body condition (24.3%), and nostril discharge (19.5%) were common among both species at ante-mortem examination. Gross lesions were frequent in livers (39.7%) and lungs (37.2%), while pneumonia (18.1%) and adhesions (13.8%) were frequent in the lungs of sheep and goats, indicating stress-related illness. Parasitic lesions, especially fasciolosis (19.3%) and hydatid cysts (8.1%) were significantly more common in sheep livers (*p* 0.05). The direct financial loss from lesions in both species was 1,077,015 ETB or 53,851 USD per year, most of which was estimated to occur from carcass bruising.

**Discussion:**

The findings indicate that reducing parasite burden and preventing carcass bruising through improved handling could significantly increase the profitability of the small ruminant meat sector in Ethiopia.

## 1 Introduction

Ethiopia has one of the largest small ruminant populations in Africa, with approximately 42.9 million sheep and 52.5 million goats (1), which account for approximately 10% of Africa’s and 4% of the world’s small ruminant population (2). This resource plays an important role in the livelihood of smallholder farmers throughout Ethiopia. A recent study estimated that the small ruminant biomass constitutes about 13% of the total livestock biomass in Ethiopia (3). There has been a rapidly increasing demand for small ruminant meat in Ethiopia, both for domestic consumption and for export trade. Nearly 86% of the country’s 93 million USD meat export revenue was made up of small ruminants in 2018/2019 (4). The small ruminant sector in Ethiopia has a large potential to meet the growing demand, and the Ethiopian government aims to further increase the production and export of small ruminant meat. However, Ethiopia’s earnings from small ruminant products are limited by problems with infrastructure, poor slaughter hygiene, high disease burden, and lack of trained personnel. Every year, significant losses occur from diseases, death of animals, and condemnation of organs and carcasses at slaughter (5). As poor health is a major contributor to low productivity of the small ruminant sector in Ethiopia, an improved understanding of the health problems is needed to support production policies and interventions.

The small ruminant meat value chain in Ethiopia includes producers, traders, handlers, and consumers of small ruminants and their products. Abattoirs are an essential node and are a source of information that can help monitor diseases and provide feedback to value chain actors based on data collected during meat inspection. The aim of meat inspection is to provide safe and wholesome meat for human consumption, which involves ante-mortem and post-mortem examination. Ante-mortem inspection identifies diseased and injured animals, while post-mortem examination reveals abnormalities and pathological processes resulting from various diseases in the carcass (6). Therefore, ante-mortem and post-mortem examinations provide useful information about the general health conditions and the presence of various diseases of the examined animals. Hence, the gathered information can help design interventions to improve the health, productivity and husbandry of the animals (7). Furthermore, abattoir data can assist in planning strategies to protect the public from zoonotic hazards (8).

A considerable number of abattoir studies have been conducted in Ethiopia. Several of these studies focused on lesions and conditions in cattle (9, 10) or on parasitic causes such as hydatidosis (11). Fewer studies focused on organs and carcass condemnation rates and associated economic losses in small ruminants in different export abattoirs in Ethiopia (5, 8). The target populations for the export abattoir-based studies were mostly limited to male sheep and goats from the lowlands that had been carefully selected and deemed suitable for slaughter at an export abattoir (and thus expected to be in better overall health condition than the general small ruminant population). A survey of slaughter animals in a major public abattoir that caters to the domestic market would give a better estimate of the existing health problems and productivity constraints of the country’s small ruminant population. Therefore, this study was carried out with the objective to improve our understanding of the health problems of sheep and goats slaughtered in Ethiopia’s largest municipal abattoir through comprehensive ante-mortem and post-mortem inspection procedures. We also estimated the direct economic loss resulting from the presence of gross lesions in organs and carcasses of sheep and goats.

## 2 Materials and methods

### 2.1 Study location

The study was conducted at the Addis Ababa Abattoir Enterprise (AAAE), which is the biggest municipal abattoir in Ethiopia, providing 85% of the meat requirements of the capital city’s residents (12). The AAAE is in the capital city of Addis Ababa and has the capacity to slaughter up to 1,000 sheep and goats per day (12). Animals are brought from the Central Highlands, passing through a hierarchy of markets. The Central Highlands region is located at 2,000–2,560 m above sea level with an average annual rainfall of 1,100 mm. Small ruminant husbandry in the Central Highlands is characterized by extensive smallholder mixed crop-livestock production system (11, 13). Small ruminants are kept mainly at communal grazing within cereal crop areas. In this system, the average flock size per household is three (sheep), or four (goats) animals (3).

### 2.2 Study design, sample size determination and sampling technique

A cross-sectional study involving ante-mortem and post-mortem inspection was conducted from January to July 2014. All age groups and both male and female sheep and goats brought from the Central Highlands to AAAE for the purpose of meat production were eligible for inclusion in the study. In order to minimize sampling multiple animals from the same herd, systematic random sampling was used by selecting every fifth eligible animal, based on the average herd size of 3–4 animals. Different lesions were expected to have different frequencies of occurrence. An expected prevalence of 50% was used in the sample size calculation, because it yielded the largest sample size among all other expected frequencies. Thus, the sample size required was calculated from expected prevalence of 50% with defined precision of 5% and level of confidence of 95% (14). Therefore, the total sample size was 768 small ruminants, including 384 sheep and 384 goats. The selected animals were identified using scotch tape, numbered using waterproof marker, and were followed for the post-mortem inspection.

### 2.3 Study methodology

All pre-slaughter examination of small ruminants was conducted by a qualified veterinarian (Tizeta Bekele) in the lairage following standard ante-mortem inspection procedures (Table 1). The age of each animal was determined based on standard methodology following the eruption of one or more incisor teeth (20). Accordingly, animals were classified as young (goats <1 year; sheep <1.5 years) or adult (goats >1 year, sheep >1.5 years). Body condition score was determined following the guidelines of the Ethiopia Sheep and Goat Productivity Improvement Program (ESGPIP) guidelines (20).

**Table 1:** Description of the clinical protocol for individual ante-mortem inspection of sheep and goats.

Clinical parameters/signs	Scores/outcomes
General behavior	0: Normal; 1: Abnormal (excessive excitability or severe depression)
Rectal body temperature (15)	0: Normal sheep: 38.3–39.9°C; normal goat: 38.5–39.7°C
	1: Higher or lower than normal
Heart rate (16)	0: Normal: sheep and goats 70–80 per minute
	1: Higher or lower than normal
Respiration rate (16)	0: Normal: sheep and goats 16–34 per minute
	1: Higher or lower than normal
Oral lesions	0: Absent; 1: Present (ulcers)
Mucous membranes	0: Normal; 1: Abnormal (pale, dry, yellow)
Eye defects	0: Absent; 1: Present
Nostril discharge	0: Absent; 1: Present (thick, white/yellow/bloody fluid from nose)
Lameness (17)	0: Normal to mild (gait is normal); 1: Moderate to severe (gait is affected)
Body condition score (15)	0: Starving, 1: Very thin, 2: Thin, 3: Moderate, 4: Fat and 5: Very fat
Skin lesions (18)	0: Absent; 1: Present (hair loss, wounds, swelling)
External parasites	0: Absent; 1: Present (lice, fleas, ticks)
Presence of tag on skin (19)	0: No tag – No visible fecal material and/or soil on the hair coat; 1: Some tag present – Some degree of matted fecal material and/or soil; 2: Medium tag – Intermediate degree of matted fecal material and/or soil; 3: Heavy tag – Large aggregates of matted fecal material and/or soil

All the small ruminants that had been examined by ante-mortem inspection were also thoroughly examined during post-mortem inspection. During this process, carcasses, and different organs (heart, lung, liver, and kidney) were thoroughly inspected by visualization, palpation and by making incisions as needed to look for the presence of abnormalities. Pathological lesions were differentiated and judged according to standard meat inspection guidelines (21, 22).

### 2.4 Estimation of direct economic loss

The analysis of direct economic loss was based on the annual slaughter rate of the abattoir, average market prices in the local market and the gross lesion frequencies of specific organs and carcass. Financial losses were calculated in terms of Ethiopian Birr (ETB) in view of the exchange rate at the time of the study (1 ETB = 0.05 USD). The annual slaughter rate was estimated from retrospective abattoir records which was 34,834 (12). Based on the current information obtained from the butcheries, the local market prices of heart, lung, liver and kidneys of sheep and goats were 2 ETB (0.1 USD), 3 ETB (0.15 USD), 5 ETB (0.25 USD), and 3 ETB (0.15 USD), respectively and that of carcass for both species of animals were 1,260 ETB (63.0 USD) per 12 kg. The direct loss was thus computed according to the formula as follows (23):

EL=ΣsrxXCoyXRoz.

Where: EL = Annual direct economic loss estimated due to organ and carcass condemnation.

Σsrk=Annualsheep/goatsslaughterrateoftheabattoir.

$$\begin{array}{l} Coy=\mathrm{Average}\;\mathrm{cost}\;\mathrm{of}\;\mathrm{each}\;\mathrm{sheep}\;\mathrm{or}\;\mathrm{goat}’s\;\mathrm{heart}/\mathrm{lung}/\\ \;\mathrm{liver}/\mathrm{kidney}\;\mathrm{and}\;\mathrm{carcass}\text{.} \end{array}$$

$$\begin{array}{l} Roz=\mathrm{Gross}\;\mathrm{lesion}\;\mathrm{frequencies}\;\mathrm{of}\;\mathrm{sheep}\;\mathrm{or}\;\mathrm{goats}/\mathrm{heart}/\\ \;\mathrm{lung}/\mathrm{liver}/\mathrm{kidney}/\mathrm{and}\;\mathrm{carcass}\text{.} \end{array}$$

### 2.5 Data analysis

Data were entered into Microsoft Excel spreadsheet (Microsoft, Redmond, WA, United States). Descriptive statistics and proportions expressed as percentages were used to analyze the data on health parameters. The Pearson chi-square test was used to assess the significance of association between species and various abnormalities/lesions, using standard statistical software (STATA IC13, StataCorp, College Station, TX, United States). A *p*-value less than 0.05 was considered statistically significant.

## 3 Results

### 3.1 Ante-mortem inspection

A total of 786 small ruminants (384 sheep and 384 goats) were subjected to ante-mortem inspection (Table 2). The presence of skin tags (medium to heavy tag; 69.1%), poor body condition (BCS < 3; 24.3%) and nostril discharge (19.5%) were the most encountered abnormalities. At least 10% of the animals also exhibited elevated heart rate (14.1%), elevated body temperature (10.5%), and increased respiratory rate (18.4%). A significantly higher proportion of sheep (22.1%) than goats (14.6%) exhibited respiratory abnormalities.

**Table 2:** Summary of abnormalities encountered during ante-mortem inspection.

Ante-mortem parameter	Number and proportion (%) of animals exhibiting parameter		
	Sheep (*n* = 384)	Goats (*n* = 384)	Total (*n* = 768)
Abnormal behavior	20 (5.2)	15 (3.9)	35 (4.6)
**Abnormal temperature**	**47 (12.2)**	34 (8.9)	**81 (10.5)**
**Abnormal heart rate**	**50 (13.3)**	**58 (15.1)**	**108 (14.1)**
Abnormal respiration rate^*^	**85 (22.1)**	**56 (14.6)**	**141 (18.4)**
Oral lesions	2 (0.5)	0 (0)	2 (0.3)
Abnormal mucus membranes	15 (3.9)	9 (2.3)	24 (3.1)
Eye defect	1 (0.3)	3 (0.8)	4 (0.5)
Nostril discharge^*^	**94 (24.5)**	**56 (14.6)**	**150 (19.5)**
Lameness/locomotion problems	2 (0.5)	2 (0.5)	4 (0.5)
**Poor body condition score**	**97 (25.2)**	**90 (23.4)**	**187 (24.3)**
Skin lesions	5 (1.3)	5 (1.3)	10 (1.3)
External parasites	3 (0.8)	3 (0.8)	6 (0.8)
**Skin tags**	**281 (73.1)**	**250 (65.1)**	**531 (69.1)**

^*^Statistically significant difference (*p* < 0.05) in occurrence of abnormality between sheep and goats. Commonly observed conditions (>10%) were highlighted in bold.

### 3.2 Post-mortem results and economic loss assessment

All animals that had been examined by ante-mortem inspection were subjected to post-mortem examination by a qualified veterinarian (Tizeta Bekele). Therefore, a total of 786 small ruminants were thoroughly examined after slaughter, including 186 young and 198 adult sheep, as well as 156 young and 228 adult goats. From the total organs examined in both species, gross lesions/diseases were most frequently detected in the livers (39.7%) and lungs (37.2%). Gross lesions were less common in hearts (3.9%), kidneys (0.65%) and carcasses (2.2%) for both species. Overall, lesions in the livers (51.0%), lungs (48.7%) and carcasses (3.6%) of sheep were significantly more common than those in in goats (28.4, 25.8, and 0.8%, respectively). The observed lesions and their respective frequencies in sheep and goats were summarized in Table 3.

**Table 3:** Summary of post-mortem lesions and associated economic losses.

Organ	Lesion	Number and proportion (%) of lesions			Annual loss (ETB)	**Annual loss (USD)**
		Sheep (*n* = 384)	Goats (*n* = 384)	Total		
Liver	Overall	196 (51.0)	109 (28.4)	305 (39.7)	69,146	3,457
	Adhesion	19 (4.9)	17 (4.4)	36 (4.7)		
	Hepatitis	38 (9.9)	30 (7.8)	68 (8.9)		
	Calcification^*^	23 (6.0)	10 (2.6)	33 (4.3)		
	Fasciolosis^*^	**74 (19.3)**	18 (4.7)	**92 (12.0)**		
	*Stilesia hepatica*	1 (0.3)	4 (1.0)	5 (0.7)		
	Hydatid cyst^*^	31 (8.1)	11 (2.9)	42 (5.2)		
	Cysticercus tenuicollis^*^	19 (4.9)	10 (2.6)	29 (3.8)		
	Abnormal coloration	3 (0.8)	0	3 (0.4)		
Lung	Overall	187 (48.7)	99 (25.8)	286 (37.2)	38,875	1,944
	Adhesions^*^	**67 (17.4)**	**39 (10.2)**	**106 (13.8)**		
	Emphysema	5 (1.3)	1 (0.3)	6 (0.8)		
	Pneumonia^*^	**88 (22.9)**	**55 (14.3)**	**143 (18.1)**		
	Hydatid cyst^*^	35 (9.1)	12 (3.1)	47 (6.1)		
	Lung worm^*^	15 (3.9)	3 (0.8)	18 (2.3)		
	*Cysticercus tenuicollis*	1 (0.3)	0	1 (0.1)		
Heart	Overall	15 (3.9)	15 (3.9)	30 (3.9)	2,717	136
	Adhesions	12 (3.1)	12 (3.1)	24 (3.1)		
	Pericarditis	2 (0.5)	2 (0.5)	4 (0.5)		
	Hydropericardium	1 (0.3)	1 (0.3)	2 (0.3)		
Kidney	Overall	3 (0.8)	2 (0.5)	5 (0.65)	679	34
	Nephritis	1 (0.3)	1 (0.3)	2 (0.3)		
	Hemorrhage	1 (0.3)	1 (0.3)	2 (0.3)		
	Calcification	1 (0.3)	0	1 (0.1)		
Carcass	Overall	14 (3.6)	3 (0.8)	17 (2.2)	965,598	48,280
	Bruising^*^	13 (3.4)	3 (0.8)	16 (2.1)		
	Hematoma	1 (0.3)	0	1 (0.1)		
Total loss					1,077,015	53,851

^*^Statistically significant difference (*p* < 0.05) in occurrence of abnormality between sheep and goats.ETB, Ethiopian Birr; USD, United States Dollar. Commonly observed conditions (>10%) were highlighted in bold.

**Figure 1:**
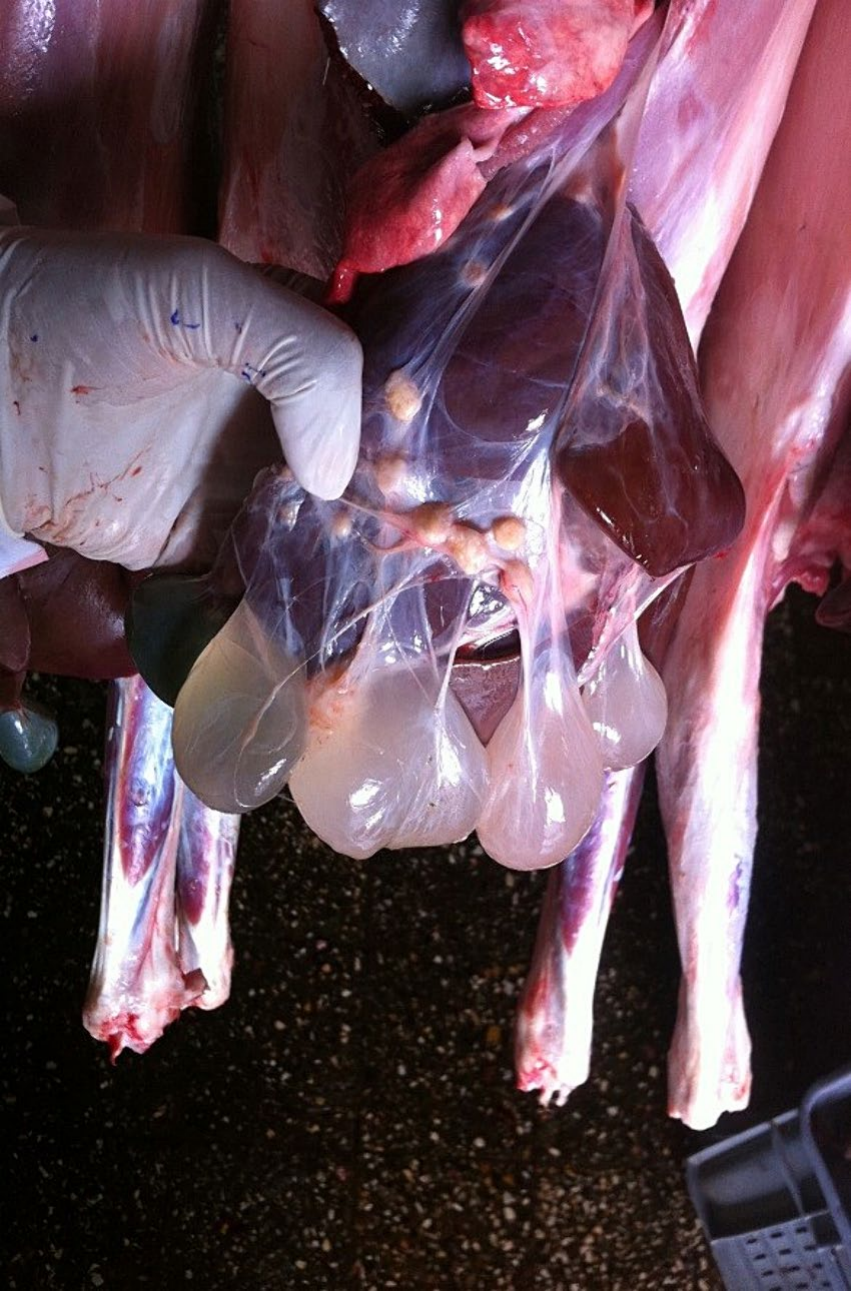
*Cysticercus tenuicollis* in the liver of sheep.

Out of the total livers inspected in both species, the most common gross pathological conditions were fasciolosis (12.0%), hepatitis (8.9%) and hydatid cysts (5.2%). There was no statistically significant difference for all types of liver lesions between age groups (*p* = 0.586) but there was statically significant difference between species (*p* < 0.001). Specifically, parasitic lesions due to fasciolosis (19.3%), hydatid cysts (8.1%) and *Cysticercus tenuicollis* (4.9%) were significantly more common in the livers of sheep (Figure 1). Liver calcification (6.0%) was also more common in sheep.

**Figure 2:**
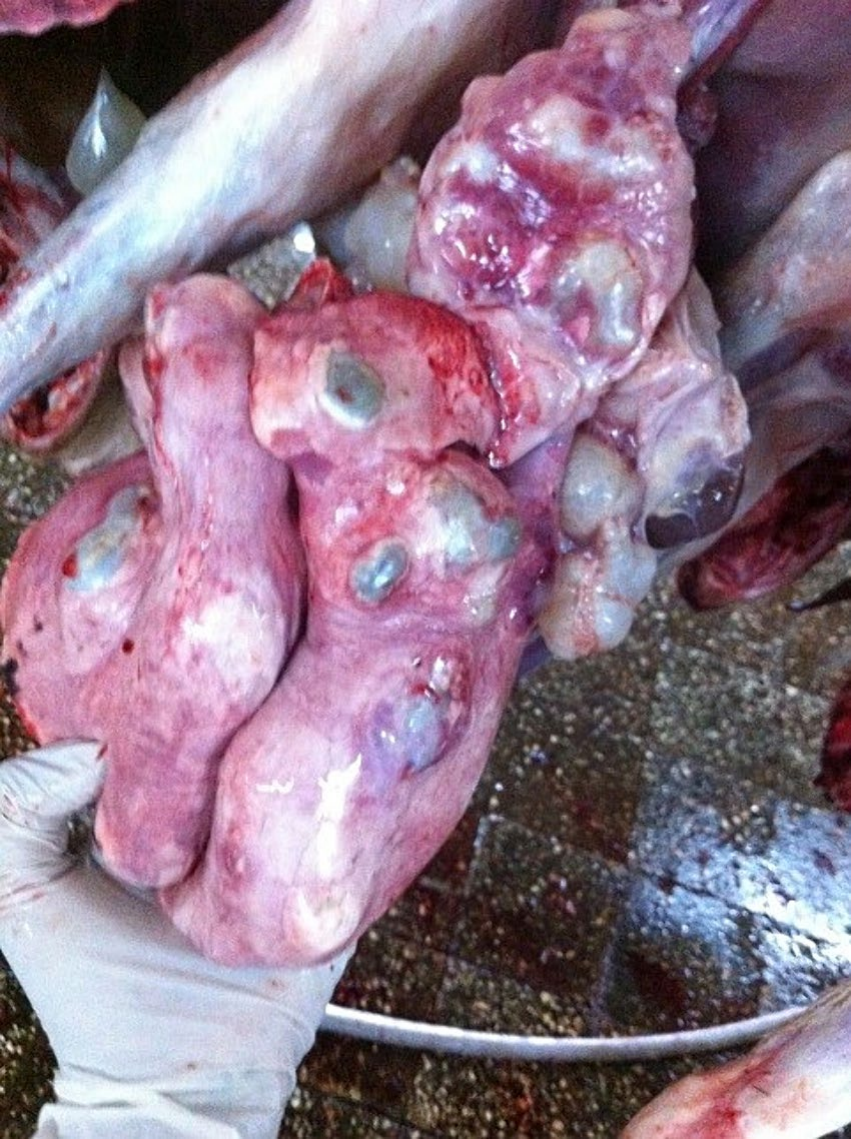
Hydatid cysts in the lung of sheep.

The major gross pathological conditions observed in the lungs were pneumonia (18.1%), adhesions (13.8%), and hydatid cysts (6.1%) in both species. There was no statistically significant difference for all causes of lung lesions between age groups (*p* = 0.781) but there was statistically significant difference between species (*p* < 0.001). Specifically, adhesions (17.4%) and pneumonia (22.9%), as well as parasitic lesions due to hydatid cysts (9.1%) and lung worms (3.9%) were significantly more common in the lungs of sheep (Figure 2).

Gross lesions in hearts were rare and included adhesions (3.1%), pericarditis (0.5%) and hydropericardium (0.3%) for both species. There was no statistically significant difference for all types of heart lesions between age groups (*p* = 0.168) and species (*p* = 0.10).

Gross pathological lesions were very rarely detected in the kidneys and included nephritis (0.3%), hemorrhage (0.3%) and calcification (0.1%) for both species. There was no statistically significant difference for all types of kidney lesions between age groups (*p* = 0.825) and species (*p* = 0.999).

Bruising (2.1%), and less commonly, hematoma (0.1%) were observed in the carcasses of sheep and goats. There was no statistically significant difference for all causes of carcass lesions between age groups (*p* = 0.114). However, the frequency of overall carcass injuries was significantly higher in sheep compared to those in goats (*p* = 0.007). Specifically, bruising was four times more common in sheep (3.4%) compared to goats (0.8%).

The annual direct economic loss from sheep and goats combined was estimated to be 1,077,015 ETB or 53,851USD per year. Most of this loss was due to carcass bruising (89.6%).

## 4 Discussion

This study investigated the presence and extent of health problems of Ethiopia’s sheep and goat population by use of a standard meat inspection methodology in the country’s largest municipal abattoir. Such data are useful to realize health problems in a specific local population. Ante-, and post-mortem inspection may then not only serve directly to provide causes for complete or partial condemnation of carcasses, but they also deliver data for supervision of the food chain and as basis for subsequent interventions. Therefore, results of this study will be very useful to stakeholders and all value chain actors in designing and prioritizing interventions. Knowledge gaps were also identified, which will assist in the design of future research activities related to the small ruminant meat industry.

The present findings indicated that livers had the highest proportion of gross lesions with an overall lesion prevalence of 39.7%. This finding is comparable to studies from export abattoirs in Ethiopia, where overall liver condemnation rates for small ruminants were in the range of 32.4 to 46.7% (5, 8). However, this study detected fasciolosis in 19.3% of all sheep livers examined, which is a much higher rate than those reported from the export abattoirs, which were in the range of 4.7 to 6.9% (5, 8). The reason for the higher rate of fasciolosis in this study compared to those from the export abattoirs can be differences in husbandry practices (i.e., application of anthelminthics) or differences in agro-ecologies. As sheep and goats acquire the infection when they consume the infectious stage of the parasite from marshy pastures, animals originating from moist highland areas (target population for present study) might be at a higher risk compared to those coming from the dryer lowlands (target population for export abattoir-based studies). A field-based study in 1993 estimated the annual economic loss associated with ovine fasciolosis in the Ethiopian highlands to be 48.4 million Ethiopian Birr or 2.42 million USD (24). As the highland sheep population appears to be a high-risk group for fasciolosis in Ethiopia with serious economic consequences, pasture management and snail control, along with strategic drenching with flukicide should be preferentially targeted to this population, to effectively reduce the disease burden and associated financial losses due to fasciolosis. The implementation of this comprehensive approach, however, may provide challenging in the small ruminant value chain in Ethiopia.

The prevalence of hydatidosis in both the livers (8.1%) and the lungs (9.1%) of sheep were also higher in the current study compared to those reported from the export abattoirs, with rates of 0.9–1 and 3.3%, respectively (5, 8). The high prevalence of hydatidosis detected in this study in sheep organs has important public health significance. The adult form of the parasite, *Echinococcus granulosus*, is a small tape worm of dogs (16), but its occurrence and infection dynamics in the Ethiopian dog population is not well known. The larval stage, referred to as hydatid cyst, is found in sheep and goats and in many other intermediate hosts including humans (16). Studies indicated that human hydatidosis is prevalent in different regions of Ethiopia (25, 26), however more studies are needed to determine its prevalence and to delineate high risk areas in the country. Larval tapeworm infections have been reported to be common in the Ethiopian highland sheep population (25, 27) due to conditions that perpetuate the life cycle of the parasite, including: (1) lack of deworming of dogs; (2) stray dogs and foxes have access to offal; (3) presence of freely roaming dogs on grazing land and (4) backyard slaughter of sheep. Dog populations are relatively scarce in the lowlands, which may explain the lower prevalence of hydatidosis reported from export slaughterhouses that source their animals from the lowlands. Prevention of larval tapeworm infection in sheep would entail controlling tapeworm infection in dogs, stray dog control, and preventing dogs from accessing sheep carcasses.

In this study, sheep had a higher proportion of parasitic lesions than goats, and lesions due to fasciolosis, hydatid cysts and *Cysticercus tenuicollis* were significantly more common in the livers of sheep. This finding is in agreement with another study conducted at Addis Ababa abattoir among sheep and goats, which reported an overall higher prevalence of hydatidosis in sheep (19.9%) than in goats (16%) (11). Another study in Tanzania also reported higher prevalence of hydatidosis in sheep than in goats (28). The higher infection rate of sheep may be due to differences in feeding habits. Sheep are grazers while goats are browsers, therefore sheep have a higher chance of being exposed to the parasite (11).

Parasites such as *Stilezia hepatica*, *Cysticercus tenuicollis* and lungworms were also found in target organs, but their prevalence was less than 5% in this survey. These parasites do not have public health importance; the direct economic losses they cause are related to organ condemnation due to aesthetic reasons (8). The indirect economic losses related to these parasites could be several magnitudes higher compared to the direct losses due to organ condemnation, because infection with these parasites significantly reduces the animal’s overall productivity (16). Therefore, reducing the disease burden from these parasites would have great economic benefits.

In this study, parasitic lesions were most commonly detected in the livers and lungs of both sheep and goats. This can be explained by the life cycle of parasites such as *Echinococcus*, whose migrating oncospheres enter the capillaries of these two organs first, before any other organ is involved (29). Lesions were detected in 37.2% of the lungs in this study for both species, which is comparable to the findings from an export slaughterhouse (41.7%) (8), with pneumonia being the most common lung lesion in the present study (18.1%) and in the above-mentioned export abattoir study (26.4%). Respiratory disorders were also commonly noted during the pre-mortem examination. Other studies have also found a high prevalence of respiratory disease in small ruminants in Ethiopia (30). The high rate of respiratory illness in small ruminants in different source populations indicates a widespread problem. In Ethiopia, conditions during transport to the abattoir are stressful for the animals, as they walk long distances or are transported in overcrowded trucks not designed for animal transport, face inclement weather, and are not offered food, feed, and rest along the way (31). These stressful conditions are known to play a predisposing role in the development of respiratory illnesses that include severe diseases such as ovine pasteurellosis, peste des petits ruminants (PPR) and contagious caprine pleuropneumonia (16, 32). Considering the high morbidity and mortality associated with these respiratory diseases, the overall economic loss from respiratory illnesses could be very substantial to the small ruminant meat industry in Ethiopia. Therefore, reducing stressful conditions, particularly during transport, could have great financial benefits to the small ruminant value chain.

The total direct economic loss associated with gross lesions was estimated to be 1,077,015 ETB or 53,851 USD per year in this study. This estimate is significantly lower than the losses calculated from commercial export abattoirs, which estimated losses at a magnitude of 300,000 to 400,000 USD per year (5, 8). The reason for this difference is that the current study used local market prices for the calculation, which are considerably lower than the international market prices. The total economic loss, however, could be much higher, because there are several indirect losses as indicated above, such as illnesses that reduce productivity, losses from poor carcass condition, and mortality on the farm and during transport, which were not included in the calculation.

Most of the direct economic loss was estimated to occur from carcass bruising (89.6%). Other studies also reported high losses due to carcass bruising (5, 8). For example, a study reported a bruising rate of 10.7% and associated loss of 13,016 ETB (651 USD) during the slaughter of 1,125 small ruminants in an export abattoir in Ethiopia. Bruising occurs during transport and handling, due to excessive use of sticks, improper transport vehicles, rough handling, and slaughter without stunning (8, 15). These reports consistently indicate that serious direct economic losses occur both from the domestic and from international markets due to carcass bruising. For example, a study conducted at a large public abattoir in Ethiopia on sheep and goats reported poor handling of animals, including beating of the body (87.7%), pushing (57.9%) and pulling (49.1%) of the animals by their handlers (15). These handling practices often resulted in animal distress and falls, which in turn result in carcass bruising. Therefore, improved handling of animals during transport and slaughter (for example, by training of stakeholders on proper handling of animals) could significantly increase the profitability of the small ruminant meat sector in Ethiopia. In consultation with the abattoir management and workers, we developed guidelines on improving animal welfare along the small ruminant value chain in Ethiopia (15).

Lastly, the presence of skin tags and poor body condition were common findings in both sheep and goats in this study and others (5, 8, 15). These problems are complex as they relate to poor nutrition and husbandry practices overall along the value chain, but they will need to be addressed for long-term growth of the small ruminant sector.

## 5 Conclusion

This study highlighted some of the major health problems of Ethiopia’s sheep and goat population by use of a standard meat inspection methodology. Results indicated that a significant proportion of livers and lungs (particularly of sheep) had gross lesions due to parasites, some of which had public health significance. Respiratory abnormalities and pneumonia were common in both species indicating stress-related illness, which could be a cause of substantial economic losses. Most of the direct economic loss was estimated to occur from carcass bruising, suggesting that improved handling of animals could significantly increase the profitability of the small ruminant meat sector in Ethiopia.

## Data availability statement

The raw data supporting the conclusions of this article will be made available by the authors, without undue reservation.

## Ethics statement

The animal study was approved by Institutional Research Ethics Committee of the International Livestock Research Institute (Ref. No. IREC-2013-03). The study was conducted in accordance with the local legislation and institutional requirements.

## Author contributions

TB: Conceptualization, Data curation, Investigation, Methodology, Writing – original draft. BS: Data curation, Formal analysis, Project administration, Writing – review & editing. AH: Investigation, Methodology, Supervision, Writing – review & editing. RF: Conceptualization, Funding acquisition, Methodology, Project administration, Resources, Supervision, Validation, Writing – review & editing. MB: Conceptualization, Project administration, Resources, Supervision, Validation, Writing – review & editing. DR: Funding acquisition, Project administration, Resources, Supervision, Writing – review & editing.
